# PRIM1 promotes the proliferation of hepatocellular carcinoma cells *in vitro* and *in vivo*

**DOI:** 10.7150/jca.47870

**Published:** 2020-09-23

**Authors:** Jinqun Jiang, Yuzhu Zhang, Rui Xu, Liping Ren, Junqian Chen, Hai Lu

**Affiliations:** 1 The Second Affiliated Hospital of Guangzhou University of Chinese Medicine , Guangzhou, Guangdong Province 510282 P.R. China;; 2 The Second Clinical College of Guangzhou University of Chinese Medicine, Guangzhou, Guangdong Province 510282 P.R. China;; 3 Department of Clinical Laboratory, Yuebei People's Hospital, Shaoguan, Guangdong Province 512026 P.R. China;; 4 Department of Breast Disease, Guangdong Provincial Hospital of Chinese Medicine, Guangzhou University of Chinese Medicine, Guangzhou, Guangdong Province 510282 P.R. China.

**Keywords:** PRIM1, Liver cancer, Lentivirus, Transfection

## Abstract

PRIM1 plays an important role during oncogenesis, however it has never been reported in liver cancer, and thus our objective is to explore the role of PRIM1 in liver cancer. We selected RNAseq data of 50 paired liver cancer samples from the Cancer Gene Atlas (TCGA), and then bioinformatics methods and Mann-Whitney U test were used to analyze the correlation between PRIM1 and the clinical pathological stage of liver cancer. Quantitative polymerase chain reaction (QPCR) was used to detect mRNA expression of PRIM1 in BEL-7404, BCL-7402, HepG2 and SMMC-7721 cell lines. LV-PRIM1-RNAi was transfected into BEL-7404 and SMMC-7721 cells by lentivirus, and then Celigo imaging cytometer, Caspase3/7 Assay, flow cytometry and MTT assay were used to detect the proliferation and apoptosis of BEL-7404 and SMMC-7721 cells with ≥50% gene reduction rate after lentivirus transfection detected by QPCR. BEL-7404 and SMMC-7721 carrying PRIM1 gene were used for oncogenesis *in vitro* to observe the weight and fluorescence intensity of the tumor. Bioinformatics method was used to obtain the information about PRIM1 gene, and the correlation between PRIM1 and clinical pathological stage of liver cancer was analyzed by Mann-Whitney U test. QPCR results showed that PRIM1 was expressed in BEL-7404, BCL-7402, HepG2 and SMMC-7721 cell lines, which was highest in BCL-7404 cell line. Celigo imaging cytometer, Caspase3/7 Assay, flow cytometry and MTT assay showed that the proliferative ability of BEL-7404 and SMMC-7721 were decreased after LV-PRIM1-RNAi transfection. Furthermore, the weight and the fluorescence intensity of the tumors *in vitro* formed by LV-PRIM1-RNAi cells on SCID mice were decreased. So, interference of PRIM1 expression can inhibit the proliferation of BEL-7404 and SMMC-7721 cells, as well as induce the apoptosis of liver cancer cells.

## Introduction

Hepatocellular carcinoma (HCC) is the 5^th^ malignant tumor 1 and 3^rd^ fatal malignant tumor in the world 2. There are 500,000 new liver cancer cases worldwide due to hepatitis B, hepatitis C and alcoholic liver disease 3. The mechanisms of the development and progression of hepatocellular carcinoma (HCC) are complicated and regulated genetically and epigenetically 4-6. Although a huge progress has been made on the research of HCC 7-9, there is only an elemental understanding of its molecular pathogenesis.

PRIM1 is a heterodimer containing a large subunit and a small subunit 10, which can synthesize RNA primer for Okazaki fragment during the discontinuous DNA replication 11. PRIM1 is the only recognized enzyme that can initiate DNA replication in eukaryotic cells 12, 13. PRIM1 expression can be induced during the whole cell cycle, which is significant during cell proliferation 14, 15. PRIM1 aberrations affect the cell cycle transition from G1 to S phase. And previous studies have proved that PRIM1 is over-expressed in various malignant tumor cells such as osteosarcoma 16, bladder carcinoma 17, breast cancer 12, and glioma 18. Wurmbach et al also reported that PRIM1 was up-regulated in HCC tissues 19; however, its specific role in development of HCC and other cancers is still undefined.

In our initial study, we analyzed the mRNA expression of PRMI-1 and evaluated the association with HCC development using data from The Cancer Genome Atlas (TCGA). We found that PRIM-1 was up-regulated in HCC tissues compared to the matched normal tissues. And its up-regulation was significantly associated with the pathological stages and pathology T stages of HCC. In this study, we hypothesize that PRIM1 modulates the proliferation of HCC cells which contributes to the progression of HCC. We investigated the molecular function of PRIM1 by knocking down the PRIM1 in HCC cell lines and examined the proliferation and apoptosis of cell lines *in vitro* and *in vivo*. In addition, the genes and signaling pathways associated with PRIM1 were also investigated by DNA microarray combined with western blotting.

Our study demonstrated that PRIM1 contributed to the proliferation of hepatocellular carcinoma cells *in vitro* and *in vivo*. PRIM1 knockdown suppresses the proliferation and promotes apoptosis of HCC cells.

## Methods

### Data retrieval from TCGA

We obtained RNAsep data of 50 paired HCC samples from TCGA (http://cancergenome.nih.gov/) and original paired data files were downloaded from the sample list based on the barcode information. Only data with complete clinical information was used for further analysis. Mann-Whitney U test was used to analyze the expression difference of PRIM1 at different TNM stage pathological stages of liver cancer.

And then Trimmed Mean of M-values (TMM) and biological coefficient of variation (BCV) were used for screening, standardization and quality control of the data. It showed that the data stability of different batches was good; the difference within the group was lower than inter-group difference, which was suitable for the following analysis. Log_2_ calculation (Cancer/Normal) was used to screen the differentially expressed genes (the filter standard was more or equal to 1, and less or equal to -1). We also applied several criteria to effectively reduce the quantity of target genes: the genes with functions which were already reported in liver cancer by existing articles were excluded; multi-transmembrane protein genes were excluded; the genes without clear annotation were excluded (for example, there is description of open reading frame); the number of cited articles about this gene in PubMed was more than 100. The data were all from reliable genetic disease databases, the finalized gene list was randomly enriched to get the final gene list for analysis.

### The endogenous expression of PRIM1 in HCC cell lines

Total RNAs of BEL-7404, BCL-7402, HepG2 and SMMC-7721 cells were collected using Trizol, and then reversely transcripted to cDNA according to the manufacturer's protocol of Promega M-MLV. Real-time PCR was used to detect the expression of PRIM1 in BEL-7404, BCL-7402, HepG2 and SMMC-7721 cells. GAPDH was used as internal control.

### The construction of lentiviruses containing shRNA against PRIM1

The shRNA sequence (CCTTGTTCCTGAAACAATT) was designed and used to target PRIM1 gene. A scrambled sequence (TTCTCCGAACGTGTCACGT) was set as a negative control construct (control RNAi) that should have no homology with the human genome. Single-stranded DNA oligos with the interfering sequence were synthesized, annealed, double digested with AgeI and EcoRI, and inserted into the lentivirus Green Fluorescent Protein (GFP) vector GV115 (GeneChem Co, Ltd, Shanghai, China) using T4 DNA Ligase (Fermentas, EL0016). The ligated vector was transfected into competent TOP10 *Escherichia coli* (TIANGEN, Cat. #CB104-03). The positive transformants were identified by PCR and validated by DNA sequencing, and the vectors with correct sequences were extracted (Qiagen non-toxic plasmid extraction kit).

### Lentivirus transfection

The vectors with correct sequences were transfected into 293T cells. After 48 h, supernatants containing the lentiviruses were harvested and the remaining cells were removed by filtering with 0.45 µm filters. The virus in the supernatant was concentrated to the target volume by centrifugation (4°C, 4,000 × g, 10 min) and the titer was finally determined by 293T cell infection assay. HCC cell lines BCL-7404 and SMMC-7721 cells were seeded in six-well plates and transfected with concentrated lentivirus in the presence of polybrene (10 μg/ml, Sigma-Aldrich, St. Louis, MO, US) according to the manufacturer's instructions. When green fluorescent protein (GFP) expression exceeded 70% in each group, cells were selected by using puromycin (5 μg/ml).

### The knockdown efficiency examined by quantitative relatime-PCR (qRT-PCR)

Total RNAs of transfected cells BEL-7404 and SMMC-7721 were extracted according to the manufacturer's protocol of Trizol extraction kit (Pufei Biotechnology Co. Ltd., Shanghai, China) and reversely transcribed to cDNA according to the manufacturer's protocol of Promega M-MLV. PCR amplification was carried out using SYBR Master Mixture (TAKARA, DRR041B). The following reaction conditions were used: 95°C for 5 sec, followed by 45 cycles of 95°C for 5 sec, 60°C for 30 sec. The 2-ΔΔCq method was applied to analyze the data (ref Livak KJ, Schmittgen TD. Analysis of relative gene expression data using real-time quantitative PCR.

### Knock-down effeciency analysed by Western blot

293T cells were coinfected by PRIM1-overexpressed plasmid and shRNA-PRIM1 expressing lentivirus according to the manufacturer's protocol of lipofectamine 2000 (Invitrogen). Cells were then lysed after 48 hours transfection. Equal amounts of protein samples (10 µg per condition) were prepared in loading buffer and boiled for 10 min, and then the boiled samples were loaded to SDS-PAGE gels and separated. Separated proteins were transferred from the gel to polyvinylidene difluoride (PVDF) membranes at 300mA for 120min. The PVDF membrane with protein samples was blocked using blocking buffer which was freshly prepared with PBS containing 5% skimmed milk powder. After blocking, anti-Flag (Sigma) and anti-GAPDH (Santa-Cruz) primary antibodies and goat anti-mouse HRP-conjugated IgG (Santa-Cruz) secondary antibodies were used and incubated with the PVDF membrane. Protein bands were detected using enhanced chemiluminescence (Pierce ECL Substrate; Thermo Fisher Scientific, Inc.), and gel analysis was performed using Image J software (version 1.8.0; National Institutes of Health, Bethesda, MD, USA).

### Cell count assay

BCL-7404 and SMMC-7721 cells from the normal control, infected with PRIM1-RNAi-Lentivirus vector and negative-control vector were seeded into 96-well plates (2000 cells/well) and incubated at 37°C in a 5% CO2 environment for 5 days. Cell count assay was performed using the Celigo image cytometer (Nexcelom Bioscience, USA). The captured cell images were analyzed by Celigo software (Nexcelom Bioscience).

### MTT assay

Cells were seeded into 96-well plates (2000 cells/well), and incubated at 37°C in a 5% CO2 environment for 1, 2, 3, 4, and 5 days. At each time point, after addition of 5 mg/mL 3-(4,5-dimethylthiazol-2-yl)-2, 5-diphenyltetrazolium bromide (MTT, 20 μL/well), cells were incubated for another 4 h and the supernatant was removed. Then, 100 μL DMSO was added to each well and cells were incubated for another 5 min with constant shaking. The absorbance (A) at 490 nm was measured using a spectrophotometric plate reader (TECAN infinite M2009MR) and cell growth curves were plotted.

### Cell apoptosis detected by flow cytometry

At the fifth day after lentivirus transfection, cells were stained with 200 μl cell suspension containing 10 μl Annexin V-APC (Cat. 88-8007, eBioscience, USA) at room temperature in the dark for 10-15 min, then flow cytometry analysis was performed on the Guava easyCyte HT flow cytometry system (Millipore).

### Cell apoptosis detected by Caspase-3/7 assay kit

Caspase-3/7 activities were measured using the Caspase-Glo® Glo Assay kit (Promega) according to the manufacturer's protocol. Cells were plated in triplicate in 96-well cell culture plates. After 3 days siRNA transfection, cells were incubated with 100 µl caspase-Glo reagent at room temperature for 30 minutes. Assays were measured by detection with a fluorescence microplate reader (TECAN infinite M2009MR).

### Tumor formation in nude mice

The BEL-7404 cells infected with PRIM1-RNAi-Lentivirus vector and negative-control vector were trypsinized, counted and resuspended in PBS. Two hundred microliters of PBS containing 2×10^7^ cells/ml were subcutaneously injected into the armpit of 4-week-old female BALB/c nude mice (n= 10 per group). The body weight and tumor volume assessment started on the 13^th^ day after cells injection and were evaluated 2 or 3 times a week. On the 24^th^ day after injection, mice were anesthetized and live fluorescence images were obtained using Lumina LT ( Perkin Elmer). Mice were then euthanized. The xenograft tumors were removed and weighed before photographed. The animal studies were approved by the Animal Ethical Committee of Southern Medical University.

### DNA microarray analysis of PRIM-1 knock-down HCC cell line

Microarray analysis of cDNA was performed according to the Techical Manual for GeneChip GeneChip primeview human (Affymetrix, 901838). Briefly, total RNA was extracted from BEL-7404 cells infected with PRIM1-RNAi-Lentivirus vector and negative-control vector. RNA integrity and purity were analyzed using an Agilent 2100 bioanalyze. Extracted RNA was converted into double-stranded cDNA with oligo-dT primer containing a T7 RNA polymerase promoter. *In vitro* transcription was performed to obtain the biotin-labeled amplified RNA (aRNA). The aRNA was purified using RNeasy Mini Kit (Qiagen) and randomly fragmented. After 16 h-hybridization at 45°C, the gene chips were washed, stained and read with Genechip Scanner 3000 (Affymetrix).

### Verification of DNA microarray using qRT-PCR

Quantitative real-time PCR (qRT-PCR) was performed to verify the results of DNA microarray results. The RNA was converted to cDNA (Promega M-MLV kit) by reverse transcription. One microliter of cDNA was amplified (Takara, cat.: RR820A) using a Roche Lightcycler 480. Thirty genes were selected for real-time PCR studies. The GAPDH gene was used as the housekeeping reference gene. The fold change was calculated using the 2△△Ct method and is presented as the fold change in the expression of pretreatment groups relative to that of the control group.

### Downstream genes regulated by PRIM1 confirmed by western blot

To verify the downstream genes regulated by PRIM1, western blot analyses were performed with anti-JUN (Abcam, ab32137), anti-EGR1 (Abcam, ab54966), anti-MET (Abcam, ab51067), anti-Wnt5a (CST, #2392), anti-PPP2R2C (Abcam, ab172086), anti-IRS (abcam, ab52167), and anti-GAPDH (Santa Cruz, SC-32233) antibodies. All experiments were repeated three times.

### Statistical analysis

SPSS12 was used to analyze the data with pairwise *t* test and analysis of variance, *P*<0.05 was considered as statistically significant.

## Results

### Bioinformatics of PRIM1

**Figure 1:**
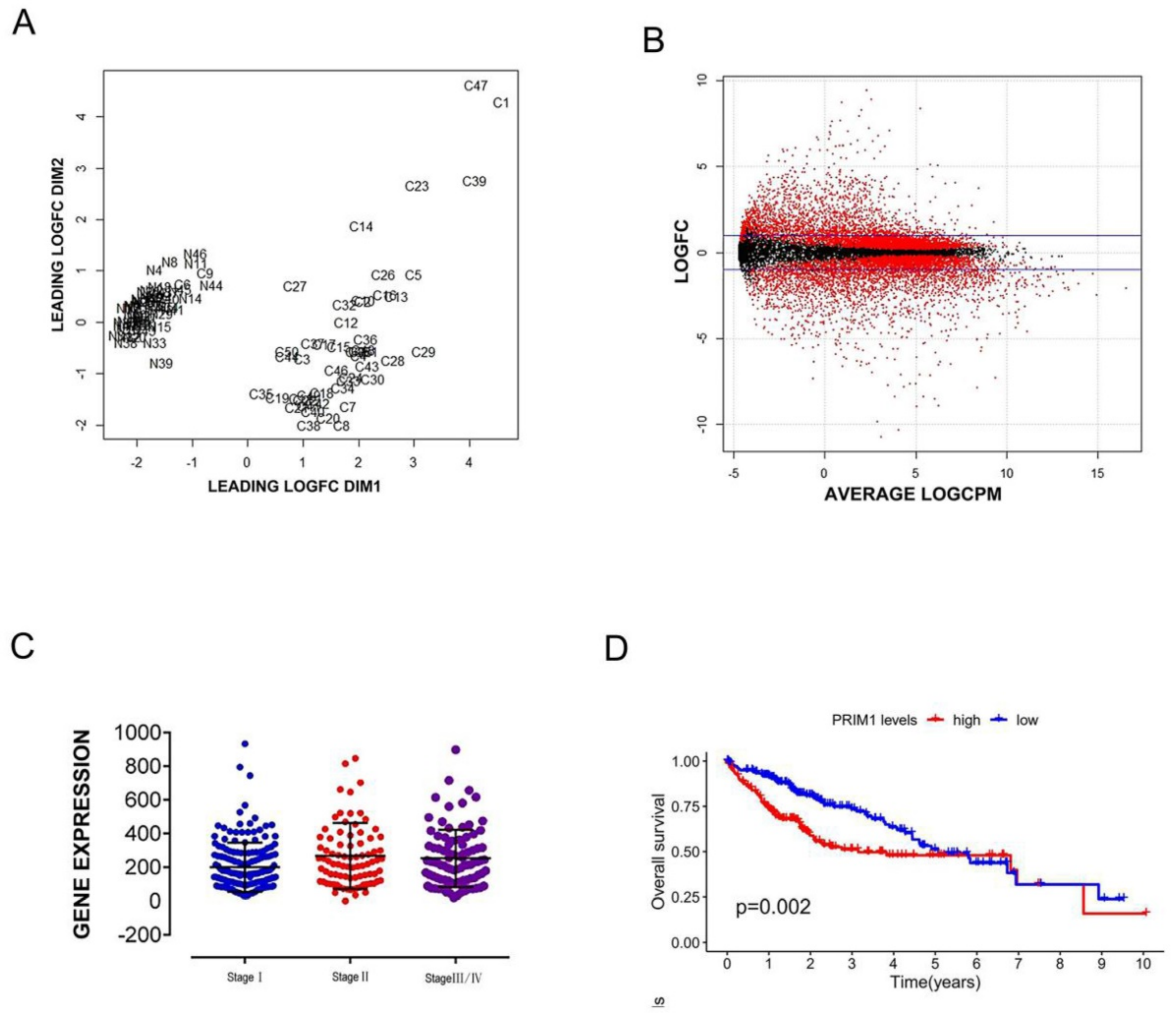
** PEIM1 gene card data from TCGA. A:** Biological coefficient of variation was used for data quality control. **B:** Get differential genes by Log_2_ (Cancer/Normal). **C:** Mann-Whitney U test was used to verify the correlation between PRIM1 and hepatocellular carcinoma. **D:** Kaplan-Meier analysis with two-sided log-rank test was performed to estimate the differences in OS between the low-risk and high-risk patients.

RNAseq data of 50 paired liver cancer samples were selected from National Cancer Institute (NCI) of America and National Human Genome Research Institute (NHGRI), and the original data files of 50 paired samples were obtained from sample list based on the barcode information of samples, there were 39 cases with complete clinical information (Table 1). PRIM1 card gene was screened by TNM standardization control, CBV quality control (Figure 1A) and log2 statistics (Figure 1B), Mann-Whitney U test was used to analyze the significant expression difference of PRIM1 on different pathological stages of liver cancer (*P*<0.05, Figure 1C). Kaplan-Meier analysis was used to analyze the OS between the low-risk and high-risk patients (*P*<0.05, Figure 1D).

### Transfection of PRIM1 with lentivirus

**Figure 2:**
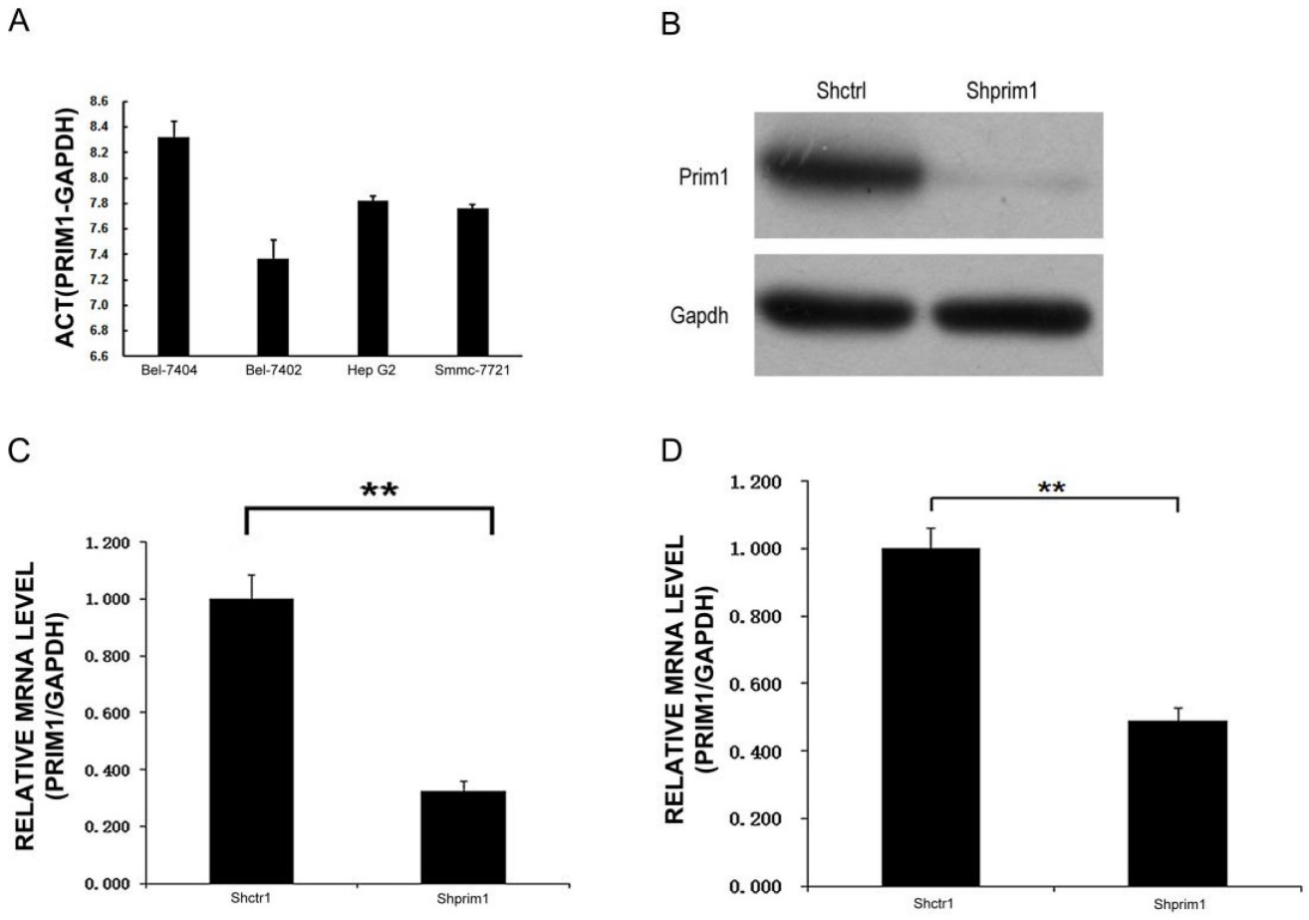
** PRIM1 expression and inhibition. A:** QPCR results showed that PRIM1 was expressed in BEL-7404, BEL-7402, HepG2 and SMMC-7721 cells. **B:** Western Blot results showed that PRIM1 was significant knockdown. **C:** PRIM1 mRNA expression was inhibited in BEL-7404 cells (67.4%); **D:** PRIM1 mRNA expression was inhibited in SMMC-7721 cells (50.9%).

QRT-PCR results showed that among the four HCC cell lines, the highest PRIM1 mRNA expression level could be seen in BEL-7404 cells, while the lowest expression of PRIM1 was detected in Bel7402 (Figure 2A). 72 hours after 293T cells were transfected by PRIM1 plasmid, more than 70% cells expressed GFP, Western Blot assay confirmed that PRIM-1 could be knockdown by shRNA-PRIM1lentivirus in 293T cells (Figure 2B). BEL-7404 and SMMC-7721 cells were chosen as models to explore the functional role of PRIM1 in HCC. QRT-PCR results showed that the after lentivirus transfection, expression of PRIM1 was significantly knockdown in both BEL-7404(Figure 2C) and SMMC-7721 cells (Figure 2D), with knockdown efficiency of 67.4% and 50.9% respectively.

### Silencing of PRIM1 inhibited HCC cells growth

**Figure 3:**
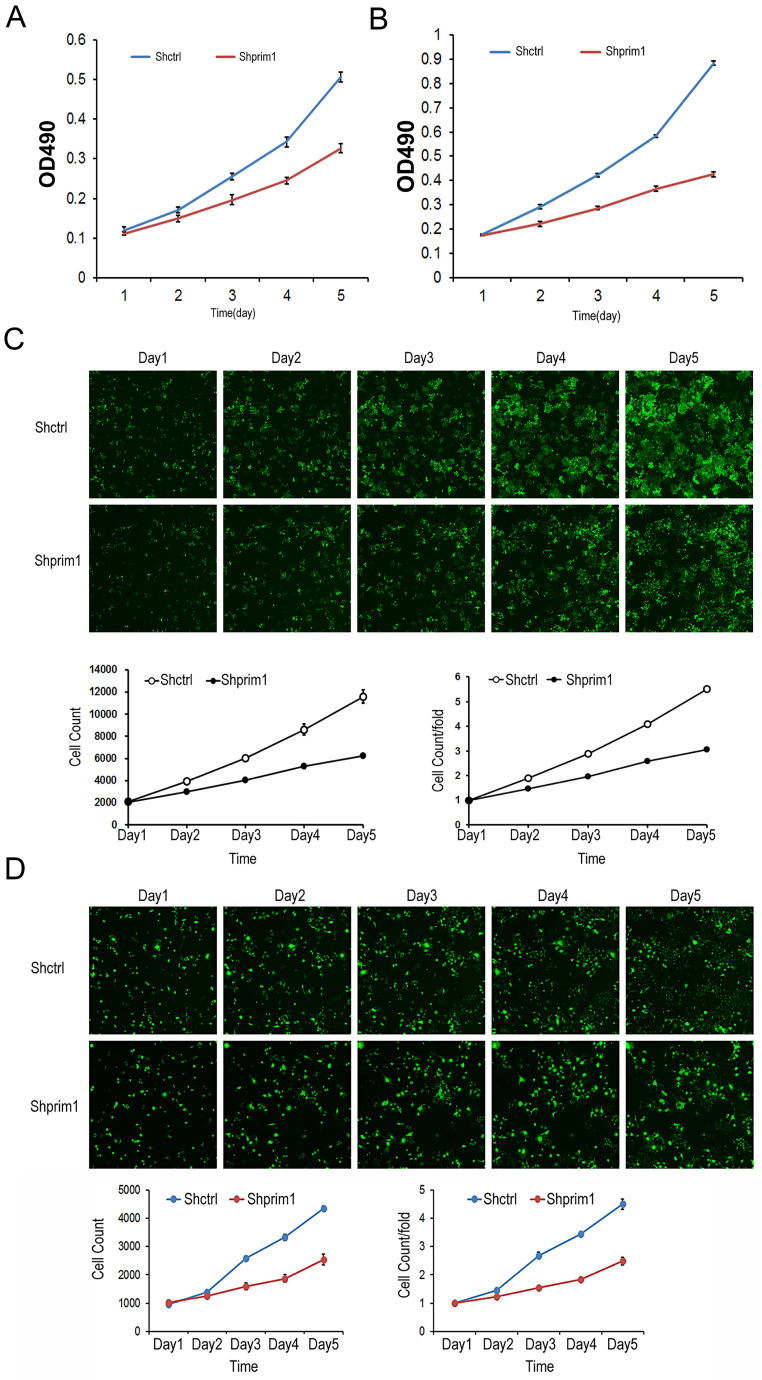
** MTT and celigo imaging cytometer after shRNA lentivirus transfection.** The proliferative rates of BEL-7404 **(A,C)** and SMMC-7721** (B,D)** was significant decreased, suggesting that PRIM1 was significantly related to the proliferative ability of BEL-7404 and SMMC-7721 cells.

The proliferation of transfected BEL-7404 and SMMC-7721 cells was detected by Celigo imaging cytometer and MTT assay. As showed in Figure 3, for both BEL-7404 and SMMC-7721 cells, the cell numbers of shPRIM1 group (cells with PRIM1-RNAi-Lentivirus vector) were significantly lower than that of shCtrl group (cells with negative-control vector). The growth curve obtained from the MTT assay indicated that the proliferative ability of shPRIM1 cells was significantly decreased when compared with that of shCtrl cells (P<0.05, Figure 3). The above results indicated that knockdown of endogenous PRIM1 by RNAi inhibited the proliferation of HCC cell lines.

### Silencing of PRIM1 induces the apoptosis of HCC cells

**Figure 4:**
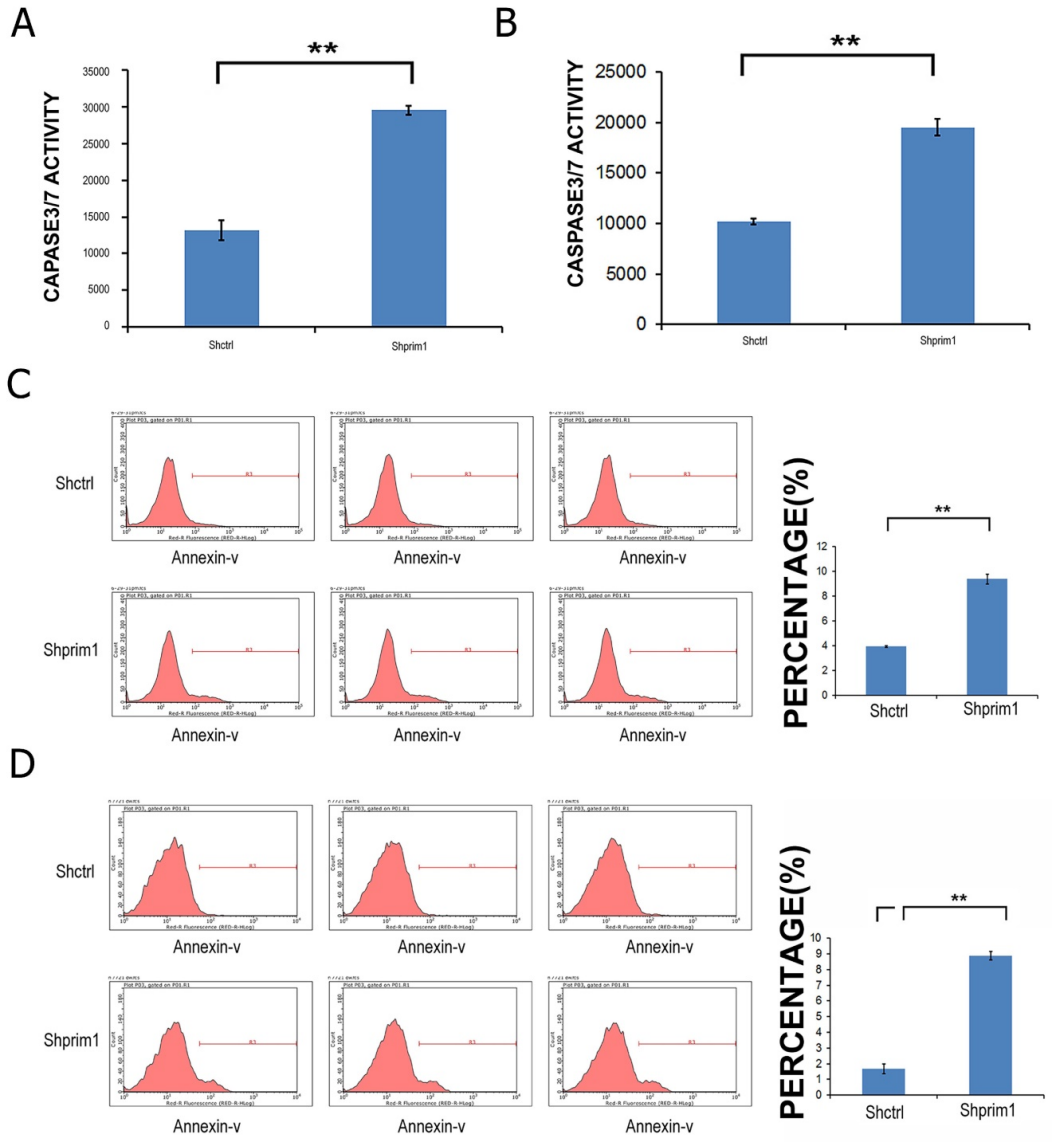
** FACS and caspase3/7 activity after shRNA lentivirus transfection.** The apoptosis of BEL-7404 **(A,C)** and SMMC-7721** (B,D)** was significant increased, suggesting that PRIM1 gene was significantly related to the apoptosis of BEL-7404 and SMMC-7721 cells.

In order to determine whether the silencing of PRIM1 induced the apoptosis of HCC cells, Annexin V-APC and caspase3/7 Assay were used to measure the apoptosis of SMMC-7721 and BEL-7404. As shown in Figure 4, after 3 days of lentivirus infection with shRNA, apoptotic cells were significantly increased, and caspase3/7 activity was significantly higher than that of control groups, suggesting that PRIM1 gene was significantly correlated with the apoptosis of HCC cells.

### PRIM1 knockdown in BEL-7404 decreased tumor growth *in vivo*

**Figure 5:**
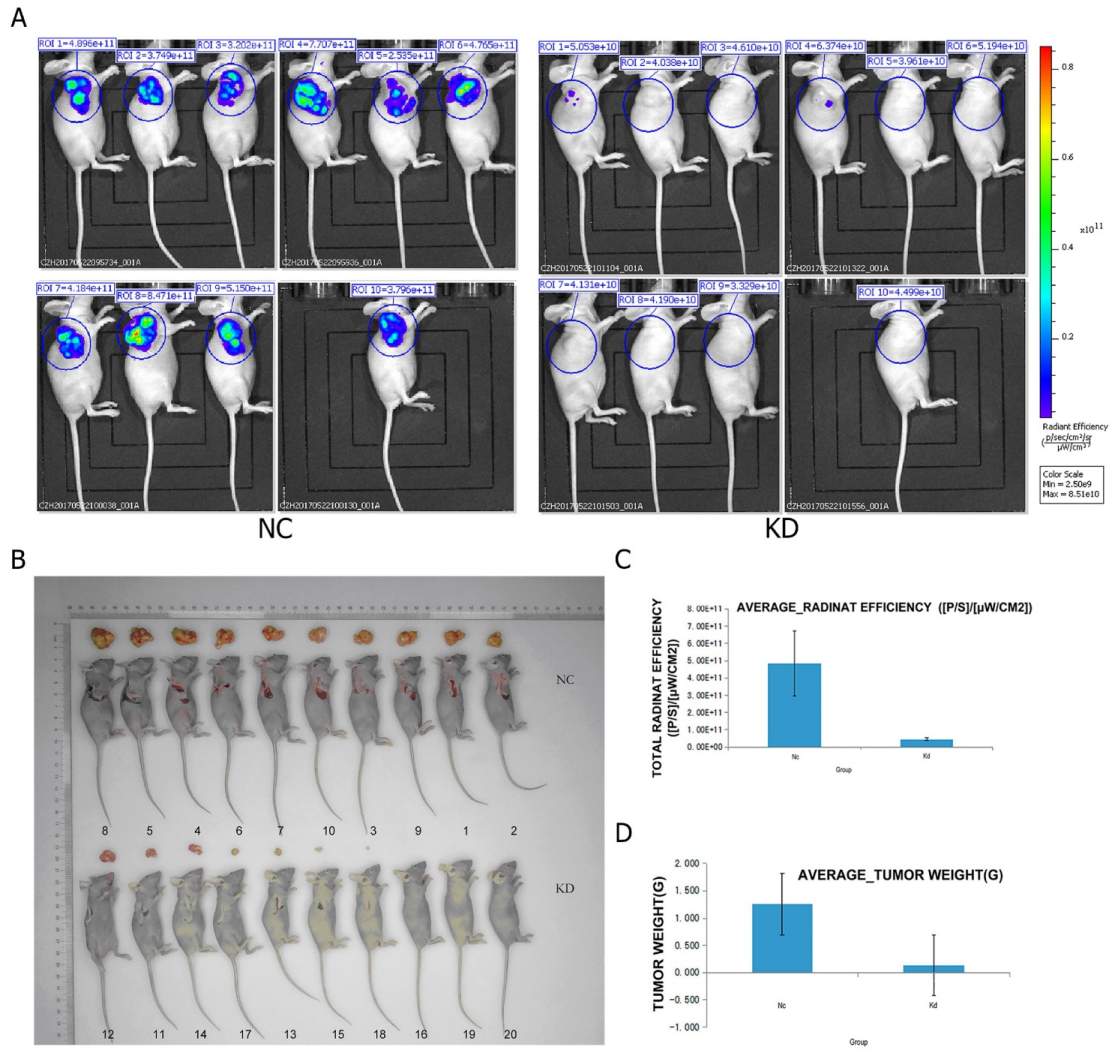
** The oncogenesis of the cells carrying LV-PRIM1-RNAiBEL-7404 in mouse. (A,D).** Compared with the control group, the tumor lesion fluorescence intensity of the experiment group was lower than the control group. **(B)** Injection of BEL-7404 cells carrying LV-PRIM1-RNAiBEL-7404 in 4-week old SCID female mice. **(C).** Compared with the control group, the average tumor lesion weight in experiment group was lower than the control group.

To further evaluate the effects of PRIM1 on the growth of HCC cell line *in vivo,* BEL-7404 cells from two groups (negative control and shPRIM1) were subcutaneously injected into BALB/c nude mice. The tumor volumes of shPRIM1 group were significantly smaller than those of negative control group from day 17 to 24 after the implantation (Figure 5). On day 24, live fluorescence images were measured using Lumina LT, and mice were then sacrificed, and the tumors were excised and weighed. Compared with negative control group, shPRIM1 group had a lower fluorescence expression (P<0.05). The weight of tumors from shPRIM1 group was significantly lower than that of negative control group (P<0.05).

### DNA microarray analysis of PRIM-1 knock-down HCC cell line

DNA microarray analysis was performed to screen out the genes that may be regulated by PRIM1. The screening criteria for significant differences in gene expression were that fold change between negative control and shPRIM1 groups must be greater than 1.5, and the p-value should be less than 0.05. DNA microarray data showed that 449 genes were up-regulated and 621 genes were down-regulated. PRIM1 genes were down-regulated with 5.35 fold-changes in shPRIM1 group. The roles of differentially regulated genes were assigned according to the IPA database.

**Figure 6:**
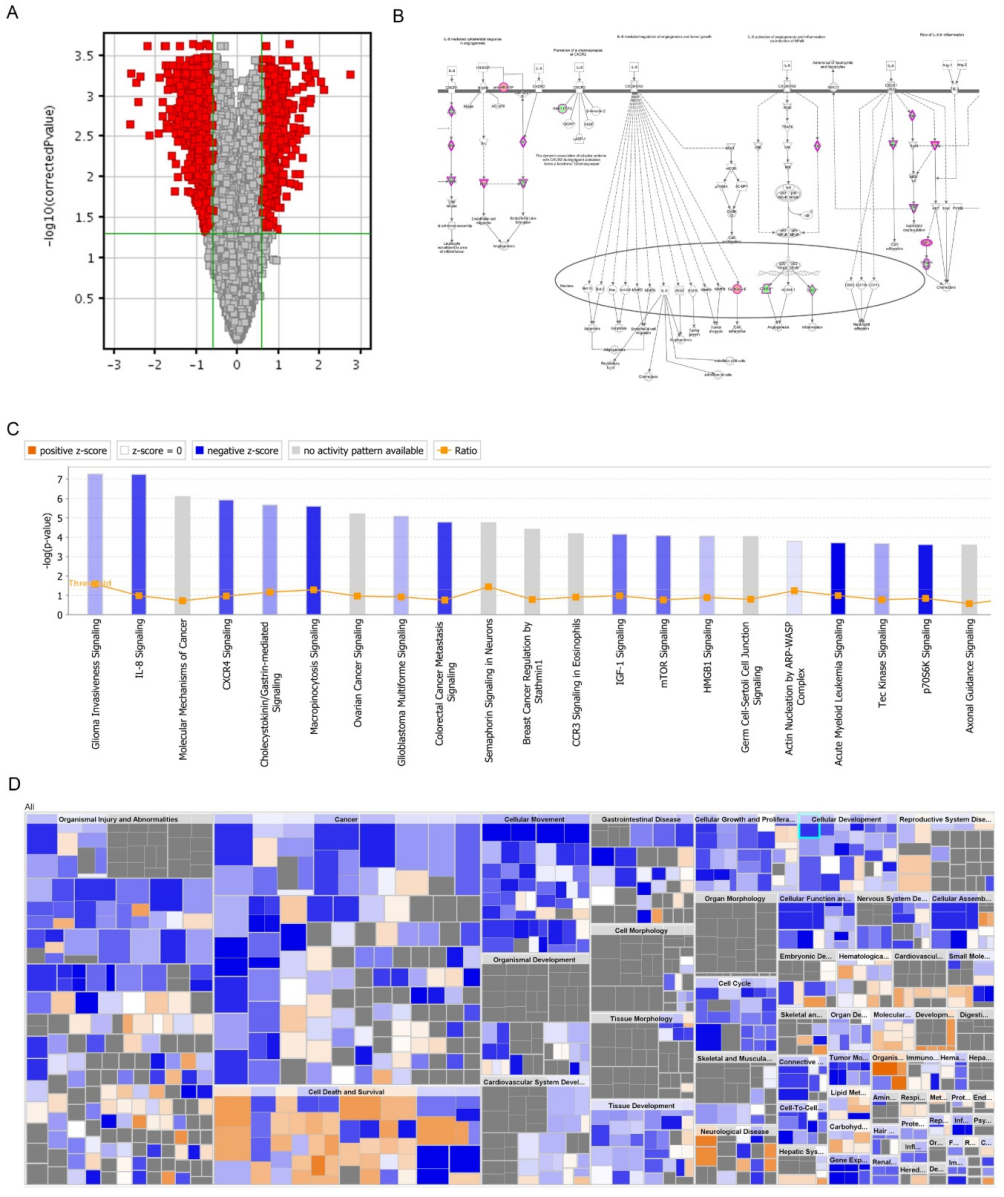
** DNA microarray analysis of PRIM-1 knock-down HCC cell line. A.** Volcano graph showed the differential gene distribution between the experimental group and the control group; **B:** In the signal path diagram, IL-8 Signaling was significantly enriches the first signal path; **C:** The classical pathway regulated by the differential gene, IL-8 Signaling, was significantly inhibited; **D:** Disease and function heat maps showed the relationship between the up- and down-regulation of differential gene expression on the inhibition of function and disease activation.

All signal pathways are sorted using -log (p-value). As shown in figure 6, the signal pathway marked in orange represents z-score >0, while the signal pathway marked in blue represents z-score <0. Z-score>2 means that the Pathway is significantly activated, and z-score <-2 means that the Pathway is significantly inhibited. Ratio represents the Ratio of the number of differential genes in this signaling pathway to the number of all genes in the signaling pathway. Figure 6 showed that differentially regulated genes were mainly enriched in the canonical pathways of glioma invasiveness signaling, IL-8 signaling, molecular mechanisms of cancer, CXCR4 signaling and et al. It was noted that IL-8 signaling was significantly suppressed in shPRIM1 group, with z-score of -2.200 and -2.132 respectively.

## Discussion

In East and South Asia the morbidity of liver cancer is highest in the world due to the prevalence of hepatitis B, which is continuously increasing 20, 21. The standard treatment for liver cancer includes surgical resection, liver transplantation and radiofrequency ablation, however at present only the early-stage patients or the patients undergo liver transplantation have 5-years survival 22, 23, thus exploring an effective method to treat liver cancer is urgent.

The function of DNA synthetase is to initiate the DNA synthesis 24, 25. DNA primase in eukaryote contains two subunits PRIM1 and PRIM2. PRIM1 is the smallest subunit of polyetherin α/primerase synthetic product in hetetotetraploid eukaryote, which individually has catalytic function of enzyme and extension function of primer 26, 27 and it plays a key role in the process of DNA synthesis initiation by synthesizing RNA primers for Okazaki fragments, however PRIM2 doesn't have the function of these enzymes 16.

During the whole cell cycle, PRIM1 mRNA expression is always regulated 28, DNA replication cannot be done without the catalytic effect of PRIM1. Thus, PRIM1 plays a critical role during oncogenesis 29. Mutations of PRIM1 cause extensive apoptosis of retinal neurons through activation of the DNA damage checkpoint and tumor suppressor p53 30.By now it has been reported in tumor cells such as bladder cancer, breast cancer and osteosarcoma 16. A large number of microarray studies have shown that abnormity of cell cycle is a sequential event 31, PRIM1 mutation can affect the transition of stage G1 to S during cell cycle. Wurmbach et al have shown that PRIM1 can be a potential marker for the early-stage liver cancer, and one important feature during the development of liver cancer is that up-regulated PRIM1 participates in the cell injury, DNA repairmen and replication.

Next, we observed that IL-8 signaling was significantly suppressed in shPRIM1 group by IPA database. IL-8, also known as CXCL8, plays an important role in the regulator of metastatic and advanced cancers. Li L et al have reported the correlation between serum IL-8 expression levels and tumor size and stage of HCC 32. IL-8 is known to be involved in stimulating HCC cell invasion and metastasis and is associated with metastasis and poor prognosis in HCC 33, 34).

## Conclusion

In our study, we analyzed the differentially expressed gene PRIM1 related to liver cancer from 50 paired tumor tissues with complete information from TCGA, QPCR results showed that PRIM1 was expressed in BEL-7404, BEL-7402, HepG2 and SMMC-7721 cells. The cell function *in vitro* and oncogenesis *in vitro* showed that interference of PRIM1 expression could inhibit the proliferation of BEL-7404 and SMMC-7721 cells. In the future study, we will screen the downstream target gene of PRIM1 by gene microarray technique to further explain the signaling transduction of PRIM1 during the development of liver cancer.

## Figures and Tables

**Table 1:** General case data included in the analysis.

	PRIM1 High	PRIM1 Low	P-value
	(N=19)	(N=20)	
**Gender**			
FEMALE	7 (36.8%)	11 (55.0%)	0.415
MALE	12 (63.2%)	9 (45.0%)	
**Age (years)**			
age <67	10 (52.6%)	9 (45.0%)	0.876
age >= 67	9 (47.4%)	11 (55.0%)	
**neoplasm_histologic_grade**			
G1	1 (5.3%)	2 (10.0%)	0.668
G2	11 (57.9%)	13 (65.0%)	
G3	7 (36.8%)	5 (25.0%)	
**Status**			
Alive	12 (63.2%)	6 (30.0%)	0.0793
Dead	7 (36.8%)	14 (70.0%)	
**pathologic_T**			
T1	7 (36.8%)	11 (55.0%)	0.52
T2	5 (26.3%)	4 (20.0%)	
T3	7 (36.8%)	5 (25.0%)	
**pathologic_N**			
N0	14 (73.7%)	16 (80.0%)	0.575
N1	1 (5.3%)	0 (0%)	
NX	4 (21.1%)	4 (20.0%)	
**pathologic_M**			
M0	14 (73.7%)	17 (85.0%)	0.279
M1	0 (0%)	1 (5.0%)	
MX	5 (26.3%)	2 (10.0%)	
**pathologic_stage**			
Stage I	6 (31.6%)	11 (55.0%)	0.273
Stage II	5 (26.3%)	4 (20.0%)	
Stage III	8 (42.1%)	4 (20.0%)	
Stage IV	0 (0%)	1 (5.0%)	

## Abbreviations

TGGA: The Cancer Genome Atlas
TGGA: The Cancer Genome Atlas
K-M: Kaplan-Meier
QPCR: Quantitative polymerase chain reaction
HCC: Hepatocellular carcinoma
TMM: Trimmed Mean of M-values
BCV: Biological Coefficient of Variation
GFP: Green Fluorescent Protein
aRNA: amplified RNA
